# PH1: An Archaeovirus of *Haloarcula hispanica* Related to SH1 and HHIV-2

**DOI:** 10.1155/2013/456318

**Published:** 2013-03-21

**Authors:** Kate Porter, Sen-Lin Tang, Chung-Pin Chen, Pei-Wen Chiang, Mei-Jhu Hong, Mike Dyall-Smith

**Affiliations:** ^1^Biota Holdings Limited, 10/585 Blackburn Road, Notting Hill, VIC 3168, Australia; ^2^Biodiversity Research Center, Academia Sinica, Nankang, Taipei 115, Taiwan; ^3^School of Biomedical Sciences, Charles Sturt University, Locked Bag 588, Wagga Wagga, NSW 2678, Australia

## Abstract

Halovirus PH1 infects *Haloarcula hispanica* and was isolated from an Australian salt lake. The burst size in single-step growth conditions was 50–100 PFU/cell, but cell density did not decrease until well after the rise (4–6 hr p.i.), indicating that the virus could exit without cell lysis. Virions were round, 51 nm in diameter, displayed a layered capsid structure, and were sensitive to chloroform and lowered salt concentration. The genome is linear dsDNA, 28,064 bp in length, with 337 bp terminal repeats and terminal proteins, and could transfect haloarchaeal species belonging to five different genera. The genome is predicted to carry 49 ORFs, including those for structural proteins, several of which were identified by mass spectroscopy. The close similarity of PH1 to SH1 (74% nucleotide identity) allowed a detailed description and analysis of the differences (divergent regions) between the two genomes, including the detection of repeat-mediated deletions. The relationship of SH1-like and pleolipoviruses to previously described genomic loci of virus and plasmid-related elements (ViPREs) of haloarchaea revealed an extensive level of recombination between the known haloviruses. PH1 is a member of the same virus group as SH1 and HHIV-2, and we propose the name *halosphaerovirus* to accommodate these viruses.

## 1. Introduction

Viruses of *Archaea* (archaeoviruses [1]) show considerable diversity and encompass novel morphotypes not seen in *Bacteria* or *Eukarya*. Relatively few have been examined in detail, partly because of the demanding growth requirements of many extremophilic *Archaea* (particularly thermophiles), and also because genetic analysis is often technically difficult compared to bacterial systems such as *Escherichia coli*. Although viruses of thermophilic *Archaea* show the most innovative capsids and replication strategies [1], the viruses of halophilic *Archaea* (haloarchaea) are of increasing attention as new isolates are found with unexpected properties. Many of the earliest reported haloviruses, including all those described before 1998, are bacteriophage-like (*Caudovirales*) with typical head-tail capsids and linear dsDNA genomes. These include groups of related viruses, such as the ΦH-like genus (ΦH, ΦCh1, and BJ1) [2–4] and the unassigned virus group comprising of HF1 and HF2 [5]. The first spindle-shaped halovirus, His1, was reported in 1998 [6, 7], and the first round virus, SH1, was described in 2003 [8, 9], and electron microscopic studies indicate that these morphotypes dominate in natural waters [10–12]. Over the last 5 years, there has been a wonderful increase in the number and types of described haloviruses, including further examples of SH1-like viruses (e.g., HHIV-2 [13]) and a range of His2-related viruses that are now classified as pleolipoviruses (e.g., HRPV-1 and HHPV-1 [14, 15]). One example of the biological novelty displayed by these archaeoviruses is the geometry of the SH1 capsid, which was found to be of a previously undescribed type, *T* = 28 dextro [16]. 

Halovirus His2 has a 16 kb dsDNA genome with terminal proteins and probably replicates via an encoded protein-primed DNA polymerase [7]. It is now known to be a pleomorphic virus, distinct from the spindle-shaped His1 [17]. When the genome sequence was first described, it was shown to be related to a cryptic plasmid (pHK2) of *Haloferax lucentense *[18, 19] and to a number of genomic loci found in several different haloarchaea. Later descriptions of haloviruses HRPV-1, HHPV-1, and several others revealed a spectrum of related viruses with very different genome structures (circular ds- and ssDNA), lengths, and replication strategies [14, 15], but they all share a similar set of capsid proteins. The mechanisms underlying the movement of the capsid genes between viruses having such different characteristics and modes of replication (protein primed versus rolling circle) remain unclear. One possibility is that such modular recombinations could be facilitated by previously described genomic loci of virus and plasmid-related elements (ViPREs) [20]. Initially, these did not appear to be simple provirus genomes in varying states of decay, but the description of pleolipoviruses with genomes much smaller than His2 and differing replication strategy can explain many of them as virus integrants. On the other hand, some of these genomic loci have expanded in length as more virus and plasmid sequences become available, and their gene homologs can be recognised in flanking sequences. For these, we retain the ViPRE epithet. From the decades of the study of bacteriophages, it is well documented that related phages frequently recombine (by both legitimate and illegitimate recombination events), giving rise to mosaic genomes with varying evolutionary histories [21, 22]. This process can be assisted by the modular arrangement of genes (e.g., capsid formation or replication) commonly found in many virus genomes [23]. The same appears true of haloviruses, such as the large recombination event evident in the comparison of halovirus HF1 and HF2 genomes [5]. More generally, there is good evidence of widespread recombination from the study of archaeal MCM helicase genes (often associated with mobile genetic elements) [24] and in the comparative genomics of archaeal caudoviruses [25].

The group of round haloviruses exemplified by SH1 has been recently expanded by the descriptions of HHIV-2 and SNJ1 [13, 26]. Members of the SH1 virus group are related by their capsid proteins but differ in genome length and replication strategy. SH1 and HHIV-2 have linear dsDNA genomes (~30.5 kb) with terminal proteins, indicative of replication by protein priming, while SNJ1 has a circular dsDNA genome of only 16.3 kb. This diversity of genome types within the same virus group closely parallels that of the pleolipoviruses described above. SH1 has been the most intensively studied, including host range, particle stability, virion structure, genome sequence, transcription mapping, transfection, and the establishment of a method of genetic manipulation [8, 13, 16, 27–31]. The aim of the current study is to describe a new member of this group, PH1. Its virological characteristics, genome sequence, and major proteins are presented and compared to other members of the SH1 virus group and to genomic loci containing related genes. For convenience, we provide the group name *halosphaerovirus* to encompass the SH1 virus group, currently consisting of SH1, PH1, HHIV-2, and SNJ1.

## 2. Results

### 2.1. Virus Isolation and Host Range

A water sample from Pink Lake, a hypersaline lake (32°00′ S and 115°30′ E) in western Australia, was screened for haloviruses by plating directly on lawns of *Har. hispanica* using overlay plates of modified growth medium (MGM) with 12% or 18% salts (w/v). A high titre of similar plaque morphology was observed (1.2 × 10^5^ PFU/mL) on the plates with 18% (w/v) salts. A novel halovirus was isolated from one of these plaques and designated PH1 based on the source (Pink Lake) and the isolating host (*Har. hispanica*).

Plaques were fully developed after two days at 37°C using overlay plates of 18% (w/v) MGM and were 1-2 mm in diameter, clear, and with ragged edges. At 30°C, plaques took three days to develop, and, at 25°C, plaques were hazy and took seven days to develop (data not shown).

The host range of PH1 was identical to that of SH1 [8]; it was unable to plaque on lawns of 12 different species belonging to six different genera of the *Halobacteriaceae* (*Haloarcula*, *Halobacterium*, *Haloferax*, *Halorubrum*, *Haloterrigena*, and *Natrialba*) but could plaque on *Halorubrum* strain CSW 2.09.4 (described in [8]), an uncharacterized Australian isolate (Table 1). 

### 2.2. Virus Purification and Particle Morphology

**Figure 1:**
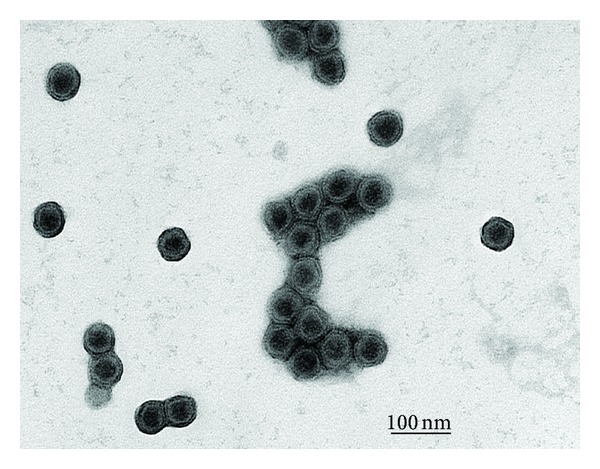
Purified PH1 virus examined by negative-stain electron microscopy. Particles were stained with 2% (w/v) uranyl acetate. Scale bar represents 100 nm.

Virus was purified by a slight modification of the method described previously for halovirus SH1 ([8] and Section 4). PH1 banded at a density of 1.29 g/mL in CsCl gradients and gave a final specific infectivity of ~5 × 10^11^ PFU/A_260_. Negative-stain electron microscopy revealed spherical particles, with an average diameter of ~51 nm (Figure 1). The capsid displayed two layers, with a compact core particle of ~43 nm in diameter. This morphology is similar to that of halovirus SH1, a closely related virus (see below) that was isolated at the same time as PH1, but from a neighbouring salt lake [8]. 

### 2.3. Single-Burst and Single-Step Growth of PH1

**Figure 2:**
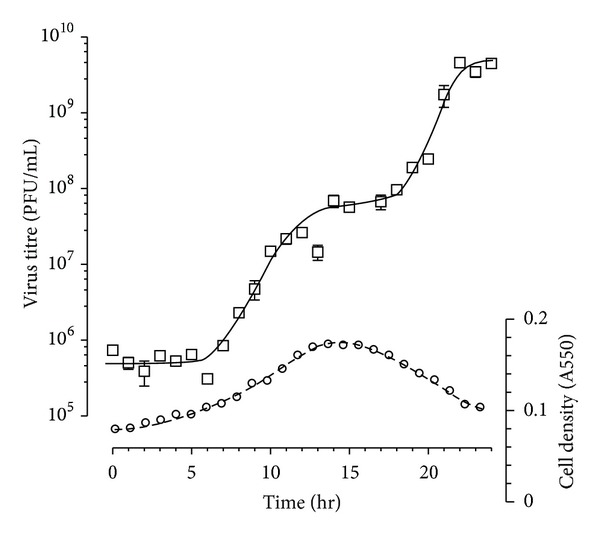
Growth curve of PH1. An early exponential culture of *Har. hispanica* in 18% (w/v) MGM was infected with virus (MOI, 50), washed to remove unbound virus, and incubated at 37°C. At regular intervals, samples were removed, the absorbance at 550 nm were measured (circles), and the number of infectious centres were determined by plaque assay (squares). Error bars represent one standard deviation of the average titre.

Around 30% of *Har. hispanica *cells could be infected by PH1, and single burst experiments [32] indicated an average burst size of 87 PFU/cell (data not shown). This value was supported by single-step growth curves, which gave burst sizes of between 50 and 100 PFU/cell. The number of infectious centres usually increased at 4–6 hr p.i., but cell lysis did not appear to begin until well after this, usually between 14 and 24 hr p.i. A representative example of a single-step growth curve for PH1 is shown in Figure 2, where the rise begins at ~6 hr p.i., and visible cell lysis begins at ~14 hr p.i. This figure shows that a second round of growth occurs at around 18 hr p.i., reflecting the initial infection of only 30% of cells, and it is during this second round of virus release that the cell density decreases most rapidly, reaching a value of about 0.1 A_550_, which is close to the initial cell density. In this example, the average burst size for the first growth step was 95 PFU/cell. 

### 2.4. Virus Stability

**Figure 3:**
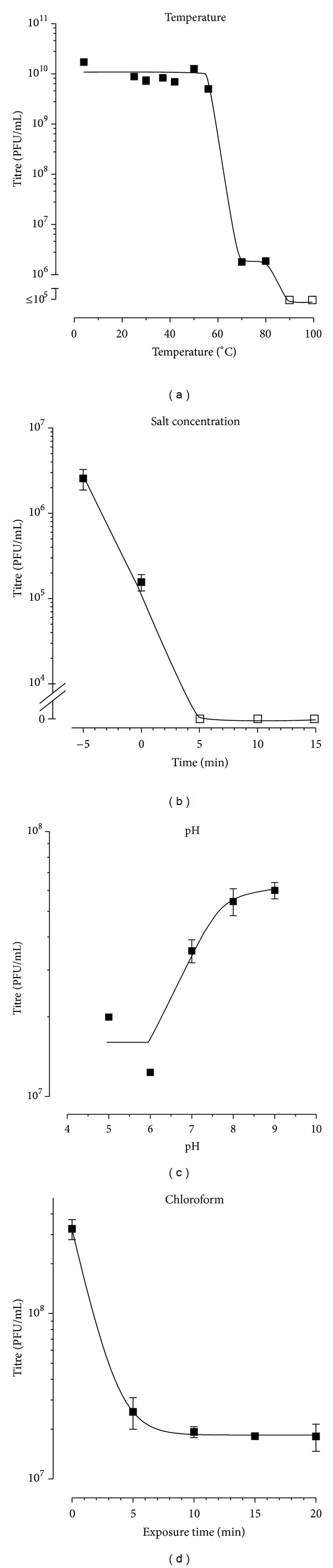
Stability of PH1 virus to various treatments and conditions. Virus preparations (infected-cell supernatants in 18% (w/v) MGM) were exposed to various conditions, after which the virus titre was determined (in duplicate) on *Har. hispanica* cells. (a) The effect of temperature. Virus was incubated for 1 hr with constant agitation at temperatures between 4 and 100°C. (b) The effect of lowered salt concentration. Virus was diluted 1 : 1,000 in double-distilled H_2_O and incubated at room temperature, with constant agitation. Samples were removed at regular intervals. (c) The effect of pH. Virus was diluted 1 : 100 in Tris-HCl buffers at the different pHs and incubated with constant agitation for 30 min. (d) The effect of chloroform. Chloroform was mixed with virus (1 : 4 ratio) and incubated at room temperature with constant agitation. Samples were removed at regular intervals. Open square symbols indicate where virus titres were undetectable. Error bars represent one standard deviation of the average titre.

The stability of PH1 virus was tested under several conditions (Figures 3(a)–3(d)). When stored in HVD at 4°C, the infectivity of PH1 remained unaltered for several months (data not shown). A thermal stability curve is presented in Figure 3(a) and shows that PH1 is stable up to 56°C above in which it rapidly loses titre. Particles were sensitive to a reduced salt environment (Figure 3(b)) and to chloroform (Figure 3(d)). PH1 was most stable between pH 8 and pH 9 (Figure 3(c)). In general, the stability of PH1 was similar to that described previously for SH1 [8].

### 2.5. PH1 Structural Proteins and Protein Complexes

**Figure 4:**
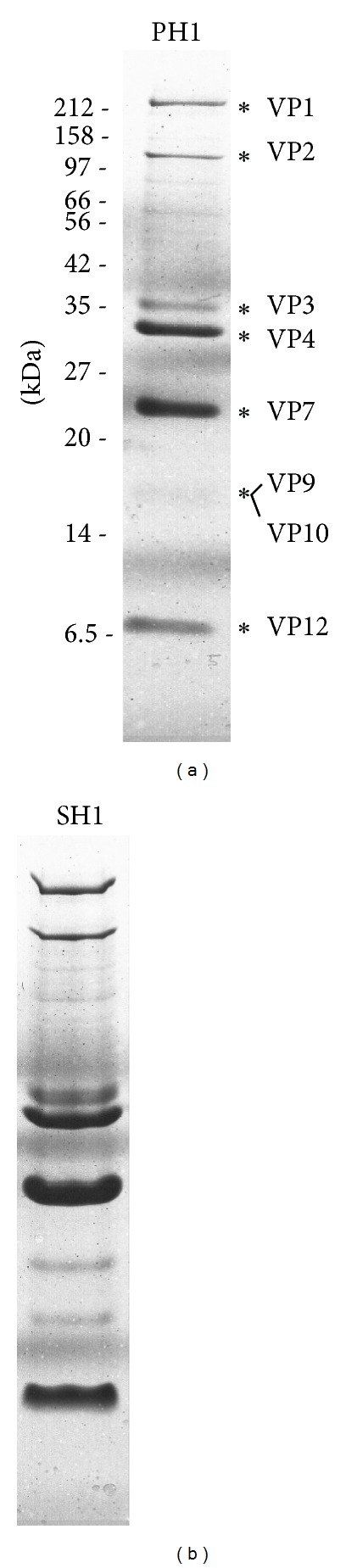
Structural proteins of halovirus PH1. Viral structural proteins were separated by SDS-PAGE on a 12% (w/v) acrylamide gel and stained with Brilliant Blue G (a). They were run in parallel with the proteins of purified SH1 virus (b). The sizes of protein standard markers are indicated on the left side (in kDa). Asterisks denote proteins bands of PH1 that were identified by mass spectroscopy. The numbering of proteins of PH1 (VP1–VP12) follows that of the SH1 homologs seen in (b) (and explained also in the text).

 The proteins of purified PH1 virus were separated by SDS-polyacrylamide gel electrophoresis, alongside the proteins of SH1 virus (Figure 4). Nine PH1 protein bands were detected, with molecular weights from 7 to 185 kDa (Figure 4(a)), and these were designated with the prefix VP and a number corresponding to the homologous protein of SH1 [8, 27] (see later). This nomenclature is consistent with that of the recently described HHIV-2 virus, a member of the same virus group [13]. The protein profile of PH1 was very similar to that of SH1 [8] and with similar relative masses of the protein bands. One notable difference was that PH1 proteins VP9 and VP10 ran closely together (calculated MWs are 16.5 and 16.7 kDa, resp.) compared to their SH1 homologs (16.5 and 16.9 kDa, resp.). It is probable, given the strong sequence similarity to SH1 (see later), that at least six additional PH1 structural proteins were unable to be visualized using our staining techniques. The potential glycosylation of virus proteins was examined by staining similar protein gels either with a periodate-acid-Schiff (PAS) stain (GelCode Glycoprotein Staining Kit, Pierce Biotechnology, USA) or the more sensitive fluorescent stain (Pro-Q Emerald 488 Glycoprotein Gel and Blot Stain Kit, Molecular Probes, USA). No glycoproteins were detected.

The major protein bands of PH1 (asterisked in Figure 4) were excised from gels, digested with trypsin, and analysed by MALDI-TOF MS, and the results are summarized in Table 2. From these data, the structural proteins VP 1–4, 7, 9, 10, and 12 were found to be specified by ORFs 12, 24, 28, 21, 20, 27, 26 and 19, respectively (see later). 

### 2.6. Characteristics of the PH1 Genome

 Nucleic acid was extracted from purified PH1 virus preparations, treated with proteinase K and incubated with various nucleases to determine the characteristics of the genome (Table 3). The PH1 genome was sensitive to dsDNA endo- and exonucleases but not to ssDNA nuclease (mung bean) or RNase A. This indicated that the PH1 genome is linear dsDNA, with free (i.e., not covalently closed) termini. Restriction endonuclease digestions of the PH1 genome gave a length of approximately 29 kb (data not shown).

**Figure 5:**
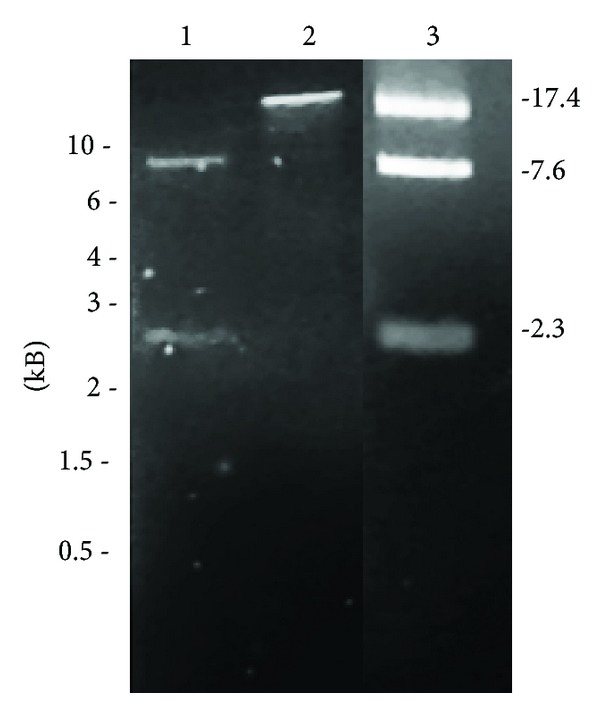
Detection of proteins bound to the termini of PH1 DNA. DNA was extracted from purified PH1 particles without proteinase K, digested with *Ase*I, then passed through a silica filter (GF/C) under conditions where proteins would bind to the filter. Lane 1: *Ase*I fragments that did not bind and were eluted. Lane 2: *Ase*I fragments that bound and were eluted only after protease treatment. Lane 3: *Ase*I digest of PH1 DNA. The positions of DNA size standards are indicated on the left side, in kb. The calculated sizes of the *Ase*I fragments of PH1 DNA in lane 3 are indicated on the right side (also in kb). The predicted 0.62 kb PH1 *Ase*I fragment was not detected.

 The presence of terminal bound proteins was examined using a silica binding assay [33]. Nonproteinase K-treated PH1 DNA was restricted with* Ase*I, and the four resulting fragments passed through GF/C filters under conditions where proteins bind firmly to the glass. As shown in Figure 5, the two internal *Ase*I fragments (2.3 and 7.6 kb) passed through the filter, but the right terminal fragment (17.4 kb) was bound, indicating it carried an attached protein. The left terminal fragment was too small (0.62 kb) and faintly staining to be detected. Further evidence was obtained by a nuclease protection assay, as previously used for SH1 DNA [31]. As shown in Table 3, exonuclease III (a 3′ exonuclease) was able to digest nonproteinase K-treated PH1 DNA, but T7 exonuclease (a 5′ exonuclease) and Bal31 (5′ and 3′ exonucleases) were unable to digest PH1 DNA unless it had been previously treated with proteinase K. This indicated that both 5′ termini of the genome had protein attached.

### 2.7. Sequence of the PH1 Genome

**Figure 6:**
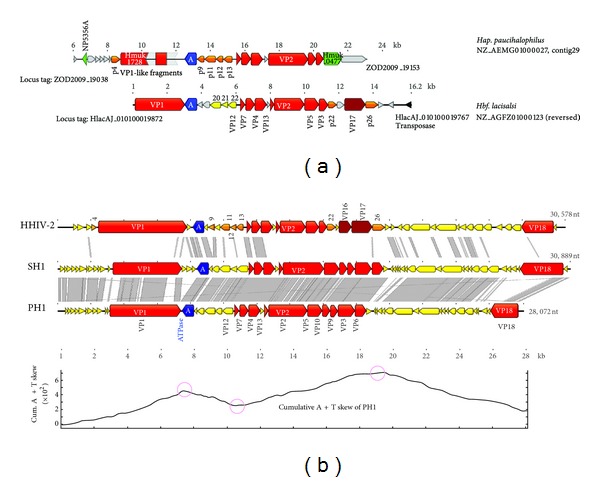
Genome alignments of haloviruses PH1, SH1, and HHIV-2, along with two related genomic loci. (a) Genomic loci of *Hap. paucihalophilus* and *Hbf. lacisalsi* that contain genes related to *halosphaeroviruses* SH1, PH1, and HHIV-2. The names and GenBank accessions for these contigs are given on the far right, and ORFs are coloured and labeled to indicate the relationships of these ORFs to those of the viruses below. The locus tag numbers for the first and last ORFs shown in each locus are given nearby their respective ORFs. In addition, grey coloured ORFs represent sequences that do not match any of the haloviruses, and green coloured ORFs represent protein sequences that are closely related to ORFs found within or very close to previously described virus/plasmid loci, so called ViPREs [20]. The scale bars shown above each contig show the position of the described region within the respective contig. (b) The three virus genomes are labeled at the left, with scale markers below (in kb) and the total length indicated at the far right. At the bottom is a cumulative AT-skew plot of the PH1 genome (http://molbiol-tools.ca/Jie_Zheng/), with inflection points circled. The grey shaded bands between the genome diagrams indicate significant nucleotide similarity (using ACT [63]). Annotated ORFs are represented by arrows, with colours indicating structural proteins (red or brown), nonstructural proteins (yellow or orange) or the packaging ATPase (blue). The names of structural protein ORFs are indicated either within the arrow (e.g., VP1) or in text nearby. The numbered, orange coloured ORFs of HHIV-2 are homologous to ORFs found in the genomic loci (probably proviruses or provirus remnants) pictured in (a).

The complete PH1 genome sequence was determined (accession KC252997) using a combination of cloned fragments, PCR, primer walking on virus DNA, and 454 whole genome sequencing (see Section 4). It was found to be 28,072 bp in length, 67.6% G + C, with inverted terminal repeat sequences (ITRs) of 337 bp. Using GLIMMER [34], manual BLAST searching at the GenBank database, and comparison to related viruses, the PH1 genome sequence was predicted to contain 49 ORFs (Table 4). Most ORFs were closely spaced or overlapping, giving a gene density of 1.74 genes/kb (average of 572 nt/gene). The cumulative AT-skew plot of the PH1 genome shown at the bottom of Figure 6 shows inflection points (circled) that are consistent with the changes in transcription direction indicated by the annotated ORFs, as has been seen in other haloviruses [7]. There are no GATC motifs in the genome, and the inverse sequence, CTAG, is strongly underrepresented with just 2 motifs (68 expected). 

When compared to the related viruses SH1 and HHIV-2 (Figure 6), the PH1 genome is seen to be of similar length and is much more closely related to SH1 than to HHIV-2 (74% and 54% nt identity, resp.). Like PH1, the genomes of SH1 and HHIV-2 lack GATC motifs, and CTAG is either absent (SH1) or underrepresented (HHIV-2). The ITRs of PH1 and SH1 share 78.5% nucleotide identity, but the PH1 ITR is longer than that of SH1 (337 and 309 nt, resp.) partly because SH1 has replaced an 18 bp sequence (gtcgtgcggtttcggcgg) found at the internal end of its left-hand ITR with a sequence at the corresponding position of its right-hand ITR that shows little inverted sequence similarity. In the PH1 ITR, this sequence is retained at both ends. Whether this difference represents a recombination event or a mistake in replication of the ITRs is unclear. An alignment of the ITRs of all three viruses identified a number of highly conserved regions (labeled 1–9 in Supplementary Figure 2, see Supplementary Material available online at http://dx.doi.org/10.1155/2013/456318). A 16 bp sequence at the termini (region 1) is conserved, consistent with the conservation seen at the termini of linear *Streptomyces* plasmids [35]. A 16 bp GC-rich sequence around the middle of the ITR (region 3) is also conserved. Shorter motifs of either C-rich (region 8) or AT-rich sequence (regions 7 and 9) are found near the internal end of the ITRs.

The grey shading between the schematic virus genomes in Figure 6(b) indicates regions of high nucleotide similarity, revealing that the differences between PH1 and SH1 are not evenly distributed but vary considerably along the length of the aligned genomes. A comparison between SH1 and HHIV-2 has been published recently [13], and since the PH1 and SH1 genomes are so similar, we will focus the following description on the differences between PH1 and SH1.

Within corresponding ORFs, there are blocks of coding sequence with low (or no) similarity. These may represent *in situ* divergence or short recombination events (e.g., indels). There are also cases where an entire ORF in one virus has no corresponding homolog in the other because of an insertion/deletion event (indel). Lastly, there are replacements, where the sequences within the corresponding regions show low or no similarity but are flanked by sequence with high similarity. The largest visible differences seen in Figure 6 are due to sequence changes within or near the long genes encoding capsid proteins VP1 and VP18. A summary of the main sequence differences between SH1 and PH1 and their locations is given in Table 5, where these divergent regions (DV) are numbered from DV1 to DV18. 

The sequences of the two viruses are close enough that in many cases the mechanism for the observed differences can be inferred. For example, DV2 is likely the result of a deletion event that has removed the SH1 ORF10 gene homolog from the PH1 genome. At either end of the SH1 ORF are almost perfect direct repeats (cggcctgac/cggcatgac) that would allow repeat-mediated deletion to occur, removing the intervening sequence. Small direct repeats leading to deletions have been described previously in the comparative analysis of *Haloquadratum walsbyi* genomes [20]. DV5 appears to be an indel, where SH1 ORFs 14–16 are absent in PH1, and an inverted repeat (AGCCATG) found at each end of the SH1 divergent region may be significant in the history of this change. The proteins specified by SH1 ORFs 14–16 are presumably dispensable for PH1 (or their functions supplied by other proteins), but a homolog of SH1 ORF14 is found also in HHIV-2 (ORF 6), and a homolog of SH1 ORF15 occurs in halovirus His1 (ORF13). Replacement regions are also common and can occur within ORFs (e.g., DV3, in capsid protein VP1) or provide additional or alternative genes (e.g., DV12 and DV13). Several divergent regions occur within the gene for capsid protein VP2, a hot spot for change because of the repetitive nature of the coding sequence, which specifies a protein with runs of glycines and many heptapeptide repeats, as described previously for SH1 VP2 [27]. DV18 is a large replacement that significantly alters the predicted length of the minor capsid protein VP18 in the two viruses (865/519 aa for SH1/PH1, resp.) and also provides SH1 with 3 ORFs that are different or absent in PH1: ORFs 52, 53, and 54. For example, a homolog of SH1 ORF52 is not present in PH1 but is present in HHIV-2 (putative protein 40). While ORF48 of PH1 shows no aa sequence homology to SH1 ORFs 53 and 54, all of these predicted proteins carry CxxC motifs, suggestive of related functions, such as DNA binding activity [36]. 

The genes of the PH1 genome are syntenic with those of SH1, and are found in similarly oriented blocks of closely spaced or overlapping ORFs that suggest that transcription is organised into operons. The programme of transcription of the SH1 genome has been reported previously [30], and the sequences in PH1 corresponding to the six promoter regions (P1–P6) determined in SH1 showed that five of these (P1–P5) are well conserved. Only the region corresponding to SH1 P6 was poorly conserved (data not shown), but this promoter is strongly regulated in SH1 and only switches on late in infection [30].

### 2.8. Structural Protein Genes

The genes coding for the major virus structural proteins of PH1 (VP 1–4, 7, 9 10, and 12) were identified by MALDI-TOF (see above), and the genes for these proteins are found in the same order and approximate positions as in the SH1 genome (Figure 6(b), red ORFs). Genes coding for minor structural proteins can be deduced by sequence comparison with SH1 so that VP5 and VP6 are likely to be encoded by PH1 ORFs 25 and 29, respectively, and VP13 and VP18 by ORFs 23 and 49, respectively. This would give PH1 a total of 11 structural proteins, the same as SH1. Of these, VP7 is the most conserved between the two viruses (98% aa identity), followed by VP4, VP9, VP3, and VP12 (91%–94% identity), VP5, VP6, and VP13 (82%–88% identity), VP2 (77%), VP1 (65%), and, lastly, the least conserved protein was VP18 (31%).

### 2.9. Genomic Loci Related to PH1 and Other Halosphaeroviruses

Clusters of halosphaerovirus-related genes are present in the genomes of two recently sequenced haloarchaea, *Hap. paucihalophilus* and *Hbf. lacisalsi *(Figure 6(a)). Neither of these regions appears to represent complete virus genome, but they retain a number of genes that share a similar sequence and synteny with the virus genomes depicted below them (Figure 6(a)). Both loci show a mixed pattern of relatedness to SH1/PH1 and HHIV-2, as indicated by ORFs of matching colour and gene name. If these genomic loci represent provirus integrants that have decayed over time, then the relationships they show suggest not only that halosphaeroviruses are a diverse virus group but also that a significant level of recombination occurs between them, leading to mosaic gene combinations. The two ORFs coloured green in the *Hap. paucihalophilus* locus (Figure 6) are related to ORFs found within or very close to genomic loci of virus/plasmid genes found in other haloarchaea, suggesting additional links between (pro)viruses [20]. 

The recently described halovirus SNJ1 carries many ORFs that have homologs adjacent to a previously described ViPRE of *Hmc. mukohataei *(from here on denoted by ViPRE_Hmuk1_), a locus that contains genes related to His2 (and other pleolipoviruses) as well as to *Haloferax* plasmid pHK2 (probably a provirus) and to two small plasmids of *Hqr. walsbyi* (~6 kb, pL6A and pL6B). The latter relationship provides wider links to other viruses because one of the PL6 genes (Hqrw_6002) has homologs in some pleolipoviruses (HPRV-3 and HGPV-1), while another gene (Hqrw_6005) is related to ORF16 of the spindle-shaped halovirus His1 [15]. After adding the SNJ1 gene homologs to ViPRE_Hmuk1_, it is extended significantly, and a close inspection of the flanking regions revealed a tRNA-ala gene at one end and a partial copy of the same tRNA-ala gene (next to a phage integrase gene) at the other end (supplementary Figure 1). Genes beyond these points appear to be related to cellular metabolism. ViPRE_Hmuk1_ is now 39379 nt in length, flanked by a 59 nt direct repeat (potential *att* sites), and includes 54 ORFs, many of which are related to known haloviruses, are virus-like (e.g., integrases, methyltransferases, transcriptional regulators, DNA methyltransferase, and DNA glycosylase), are homologs in or near other known ViPREs, or show little similarity to other known proteins. This locus also includes an ORC1/CDC6 homolog (Hmuk_0446), which could provide a replication function. 

The mixture of very different virus and plasmid genes seen within ViPRE_Hmuk1_ is remarkable, but it also reveals homologs found in or adjacent to genomic loci of other species, such as the gene homologs of *Har. marismortui* seen in the left end of ViPRE_Hmuk1_ (supplementary Figure 1). When these genes are added to the previously described *Haloarcula* locus (ViPRE_Hmuk1_), it is also extended significantly. It becomes 25,550 bp in length, encompasses genes from Hmar_2382 to Hmar_2404, and is flanked by a full tRNA-ala gene at one end and a partial copy at the other. It carries an ORC1/CDC6 homolog, an integrase, and a phiH-like repressor as well as pleolipovirus homologs. 

### 2.10. Transfection of Haloarchaea by PH1 DNA

**Figure 7:**
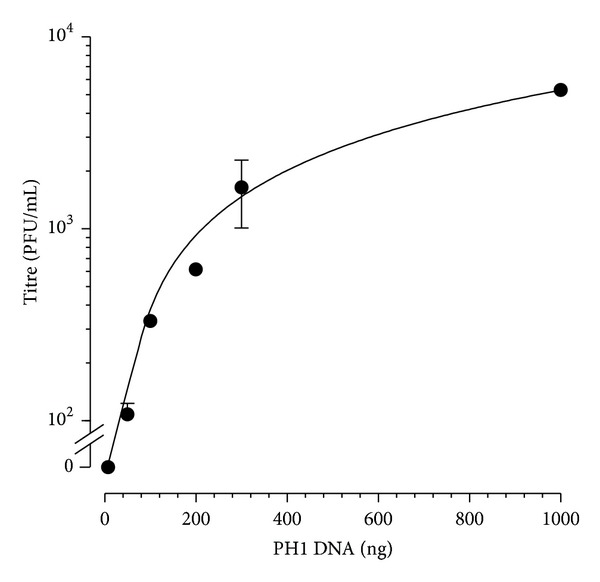
Transfection of *Har. hispanica *cells with PH1 DNA. Varying amounts of nonproteinase K-treated PH1 DNA were introduced into cells of *Har. hispanica *using the PEG method [64]. Cells were then screened for infective centres by plaque assay. Data shown is the average of three independent experiments, performed in duplicate. Error bars represent one standard deviation of the mean. If protease-treated DNA was used, no transfectant plaques were observed.

PH1 DNA was introduced into *Har. hispanica* cells using the PEG method [37], and the cells were screened for virus production by plaque assay.  Figure 7 shows the increase in transfected cells with input (nonproteinase K-treated) virus DNA. The estimated efficiency was 5.3 × 10^3^ PFU per *μ*g DNA. PH1 DNA that had been treated with proteinase K or with DNAase I (RNase-free) did not produce plaques (data not shown). PH1 DNA was found to transfect six species of haloarchaea other than *Har. hispanica*, including members of the genera *Haloarcula*, *Haloferax*, *Halorubrum*, *Haloterrigena,* and *Natrialba* (Table 6). For these experiments, the virus production from transfected cells was detected by plaque assay on indicator lawns of *Har. hispanica*. 

## 3. Discussion

PH1 is very similar in particle morphology, genome structure, and sequence to the previously described halovirus SH1 [8, 16]. Like SH1, it has long inverted terminal repeat sequences and terminal proteins, indicative of protein-primed replication. Purified PH1 particles have a relatively low buoyant density and are chloroform sensitive and show a layered capsid structure, consistent with the likely presence of an internal membrane layer, as has been shown for SH1, HHIV-2, and SNJ1 [13, 26]. The particle stability of PH1 to temperature, pH, and reduced salt was similar to SH1 [8]. The structural proteins of PH1 are very similar in sequence and relative abundance to those of SH1, so the two viruses are likely to share the same particle geometry [16]. 

Viruses that use protein-primed replication, such as bacteriophage Φ29, carry a viral type B DNA polymerase that can interact specifically with the proteins attached to the genomic termini and initiate strand synthesis [38]. Archaeoviruses His1 [7] and *Acidianus* bottle-shaped virus [39] probably use this mode of replication. However, a polymerase gene cannot be found in the genomes of PH1, SH1, or HHIV-2. DNA polymerases are large enzymes, and the only ORF of SH1 that was long enough to encode such an enzyme and had not previously been assigned as a structural protein was ORF55, which specified an 865 aa protein that contained no conserved domains indicative of polymerases [27]. The recent study of HHIV-2 showed that the corresponding ORF in this virus (gene 42) is a structural protein of the virion, and, by inference, this is likely also for the corresponding proteins of SH1 and PH1 (no homolog is present in SNJ1). Without a polymerase gene, these viruses must use a host enzyme, but searches for a viral type B DNA polymerase in the genome of the host, *Har. hispanica*, or in the genomes of other sequenced haloarchaea, did not find any matches. This argues strongly for a replication mechanism that is different to that exemplified by Φ29. An attractive alternative is that displayed by *Streptomyces* linear plasmids, which have 5′-terminal proteins and use a cellular polymerase for replication [40]. The terminal proteins are not used for primary replication but for end patching [41], and it has been shown that if the terminal repeat sequences are removed from these plasmids and the ends ligated, they can replicate as circular plasmids [42]. This provides a testable hypothesis for the replication of halosphaeroviruses and also offers a pathway for switching between linear (e.g., PH1) and circular (e.g., SNJ1) forms. Use of a host polymerase would also fit with the ability of SH1 and PH1 DNA to transfect many different haloarchaeal species [31] as these enzymes and their mode of action are highly conserved.

The growth characteristics of PH1 in *Har. hispanica*, both by single-cell burst and single-step growth experiments, gave values of 50–100 viruses/cell, significantly less than that of SH1 (200 viruses/cell) [8] and HHIV-2 (180 viruses/cell) [13]. The latter virus is lytic, whereas SH1, PH1, and SNJ1 start producing extracellular virus well before any decrease in cell density, indicating that virus release can occur without lysis. This has been most clearly shown with SH1, where almost 100% of cells can be infected [8]. Infection of *Har. hispanica* by PH1 was less efficient (~30% of cells), but the kinetics of virus production in single-step growth curves of SH1 and PH1 are similar, with virus production occurring over several hours rather than at a clearly defined time after infection. The two viruses are also very closely related and infect the same host species, so it is likely that they use the same scheme for cell exit. For both viruses, the cell density eventually decreases in single-step cultures, showing that virus infection does result in cell death. This mode of exit appears to maximise the production of the virus over time, as the host cells survive for an extended period. In natural hypersaline waters, cell numbers are usually high, but growth rates are low [12], and the high incident UV (on often shallow ponds) would damage virus DNA, so reducing the half-life of released virus particles [43]. These factors may have favoured the exit strategy displayed by these viruses, improving their chance of transmission to a new host.

The motif GATC was absent in the genomes of PH1, SH1, and HHIV-2, and the motif CTAG was either absent or greatly underrepresented. All three viruses share the same host, but GATC and CTAG motifs are plentiful in the *Har. hispanica* genome (accessions CP002921 and CP002923). Avoidance of these motifs in haloviruses His1, His2, HF1, and HF2 has been reported previously [7]. Such purifying selection commonly results from host restriction enzymes, and, in *Hfx. volcanii,* it is exactly these two motifs that are targeted [44]. One *Hfx. volcanii *enzyme recognises A-methylated GATC sites [45], and another recognises un-methylated CTAG sites (which are protected by methylation in the genome). In the current study, this could explain the negative transfection results for *Hfx. volcanii*, as the PH1 genome contains two CTAG motifs. By comparison, the SH1 genome contains no CTAG motifs and is able to transfect *Hfx. volcanii* [31].

Comparison of the PH1 and SH1 genomes allowed a detailed picture of the natural variation occurring between closely related viruses, revealing likely deletion events mediated by small repeats, a process described previously in a study of *Hqr. walsbyi* and termed repeat-mediated deletion [20]. There are also many replacements, including blocks of sequence that occur within long open reading frames (such as the capsid protein genes, VP1 and VP18). VP3 and VP6 are known to form the large spikes on the external surface of the SH1 virion, and presumably one or both interact with host cell receptors [16]. PH1 and SH1 have identical host ranges, consistent with the high sequence similarity shown between their corresponding VP3 and VP6 proteins. 

 A previous study of SH1 could not detect transcripts across annotated ORFs 1–3 or 55-56 [30]. ORFs 1 and 56 occur in the terminal inverted repeat sequence, and, in the present study, it was found that there were no ORFs corresponding to these in the PH1 genome. Given the close similarity of the two genomes, the comparative data are in agreement with the transcriptional data, indicating that these SH1 ORFs are not used. SH1 ORFs 2 and 3 are more problematic, as good homologs of these are also present in PH1. The conflict between the transcriptional data and the comparative genomic evidence requires further experimental work to resolve. The status of SH1 ORF55 has recently been confirmed by studies of the related virus, HHIV-2 (discussed above).

The pleolipovirus group has expanded dramatically in the last few years, and it now comprises a diverse group of viruses with different genome types and replication strategies. What is even more remarkable is that a similar expansion can now be seen with SH1-related viruses, which includes viruses with at least two replication modes and genome types (linear and circular). Evidence from haloarchaeal genome sequences show cases of not only provirus integrants or plasmids (e.g., pKH2 and pHH205) but also genomic loci (ViPREs) that contain cassettes of virus genes from different sources, and at least two cases (described in this study) have likely *att* sites that indicate circularisation and mobility. While there is a clear relationship between viruses with circular ds- and ssDNA genomes that replicate via the rolling circle method (i.e., the ssDNA form is a replication intermediate); it is more difficult to explain how capsid gene cassettes can move between these viruses and those with linear genomes and terminal proteins. Within the pleolipoviruses, His2 has a linear genome that contains a viral type B DNA polymerase and terminal proteins, while all the other described members have circular genomes that contain a *rep* homolog and probably replicate via the rolling circle method [14, 19]. Similarly among the halosphaeroviruses, PH1, SH1, and HHIV-2 have linear dsDNA with terminal proteins and probably replicate in the same way, but the related SNJ1 virus has a circular dsDNA genome. The clear relationships shown by the capsid genes of viruses within each group, plus the connections shown to haloviruses outside each group (e.g., the DNA polymerases of His1 and His2), all speak of a vigorous means of recombination; one that can readily switch capsid genes between viruses with radically different replication strategies. How is the process most likely to operate? One possibility is suggested by ViPREs, which appear to be mobile collections of capsid and replication genes from different sources. They offer fixed locations for recombination to occur, provide gene cassettes that can be reassorted to produce novel virus genomes, and some can probably recombine out as circular forms. For example, ViPRE_Hmuk1_ carries genes related to pleolipoviruses, halosphaeroviruses, and other haloviruses. It is yet to be determined if any of the genes carried in these loci are expressed in the cell (such as the genes for capsid proteins) or if ViPREs provide any selective advantage to the host. 

## 4. Materials and Methods

### 4.1. Water Sample

A water sample was collected in 1998 from Pink Lake (32°00′ S and 115°30′ E), a hypersaline lake on Rottnest Island, Western Australia, Australia. It was screened in the same year for haloviruses using the methods described in [8]. A single plaque on a *Har. hispanica* lawn plate was picked and replaque purified. The novel halovirus was designated PH1.

### 4.2. Media, Strains, and Plasmids

 The media used in this study are described in the online resource, The Halohandbook (http://www.haloarchaea.com/resources/halohandbook/index.html). Artificial salt water, containing 30% (w/v) total salts, comprised of 4 M NaCl, 150 mM MgCl_2_, 150 mM MgSO_4_, 90 mM KCl, and 3.5 mM CaCl_2_ and adjusted to pH 7.5 using ~2 mL 1 M Tris-HCl (pH 7.5) per litre. MGM containing 12%, 18%, or 23% (w/v) total salts and HVD (halovirus diluent) were prepared from the concentrated stock as previously described [46]. Bacto-agar (Difco Laboratories) was added to MGM for solid (15 g/L) or top-layer (7 g/L) media. 

Table 1 lists the haloarchaea used in this study. All haloarchaea were grown aerobically at 37°C in either 18% or 23% (w/v) MGM (depending on the strain) and with agitation (except for “*Har. sinaiiensis*”). The plasmids used were pBluescript II KS+ (Stratagene Cloning Systems), pUBP2 [47], and pWL102 [48]. pUBP2 and pWL102 were first passaged through *E. coli* JM110 [49] to prevent *dam*-methylation of DNA, which has been shown to reduce transformation efficiency in some strains [45].

### 4.3. Negative-Stain Electron Microscopy

 The method for negative-stain TEM was adapted from that of V. Tarasov, described in the online resource, The Halohandbook (http://www.haloarchaea.com/resources/halohandbook/index.html). A 20 *μ*L drop of the sample was placed on a clean surface, and the virus particles were allowed to adsorb to a Formvar film 400-mesh copper grid (ProSciTech) for 1.5–2 min. They were then negatively stained with a 20 *μ*L drop of 2% (w/v) uranyl acetate for 1-2 min. Excess liquid was absorbed with filter paper, and the grid was allowed to air dry. Grids were examined either on a Philips CM 120 BioTwin transmission electron microscope (Royal Philips Electronics), operating at an accelerating voltage of 120 kV, or on a Siemens Elmiskop 102 transmission electron microscope (Siemens AG), operating at an accelerating voltage of 120 kV.

### 4.4. Virus Host Range

 Twelve haloarchaeal strains from the genera *Haloarcula*, *Halobacterium*, *Haloferax*, *Halorubrum,* and *Natrialba* (Table 1), Thirteen natural *Halorubrum* isolates (H. Camakaris, unpublished data), and five uncharacterized haloarchaeal isolates from Lake Hardy, Pink Lake (in Western Australia) and Serpentine Lake (D. Walker and M. K. Seah, unpublished data), were screened for PH1 susceptibility. Lysates from PH1-infected *Har. hispanica* cultures (1 × 10^11^ PFU/mL) were spotted onto lawns of each strain (using media with 12% or 18% (w/v) MGM, depending on the strain) and incubated for 2–5 days at 30 and at 37°C.

### 4.5. Large Scale Virus Growth and Purification

Liquid cultures of PH1 were grown by the infection (MOI, 0.05) of an early exponential *Har. hispanica* culture in 18% (w/v) MGM. Cultures were incubated aerobically at 37°C, with agitation, for 3 days. Clearing (i.e., complete cell lysis) of PH1-infected cultures did not occur, and consequently cultures were harvested when the absorbance at 550 nm reached the minimum, and the titre (determined by plaque assay) was at the maximum, usually ~10^10^–10^11^ PFU/mL. Virus was purified using the method described previously for SH1 [8, 31], except that after the initial low speed spin (Sorvall GSA; 6,000 rpm, 30 min, 10°C); virus was concentrated from the infected culture by centrifugation at 26,000 rpm (13 hr, 10°C) onto a cushion of 30% (w/v) sucrose, in HVD. The pellet was resuspended in a small volume of HVD and loaded onto a preformed linear 5%–70% (w/v) sucrose gradient, followed by isopycnic centrifugation in 1.3 g/mL CsCl (Beckman 70Ti; 60,000 rpm, 20 hr, 10°C). The white virus band occurred at a density of 1.29 g/mL and was collected and diluted in halovirus diluent (HVD, see Section 4), and the virus pelleted (Beckman SW55; 35,000 rpm, 75 min, 10°C) and resuspended in a small volume of HVD and stored at 4°C. Virus recovery at the major stages of a typical purification is given in supplementary Table 1. The specific infectivity of pure virus solutions was determined as the ratio of the PFU/mL to the absorbance at 260 nm.

### 4.6. PH1 Single-Step Growth Curve

 An early exponential phase culture of *Har. hispanica* grown in 18% (w/v) MGM was infected with PH1 (MOI, 50). Under these conditions the percentage of infected cells was approximately 30%. After an adsorption period of 1 hr at 37°C, the cells were washed three times with 18% (w/v) MGM (at room temperature), resuspended in 100 mL 18% (w/v) MGM (these methods ensured the removal of all residual virus), and incubated at 37°C, with shaking (100 rpm). Samples were removed at hourly intervals for measurements of absorbance at 550 nm and the number of infective centres. Immediately after sampling, titres were determined by plaque assay with an indicator lawn of *Har. hispanica*. Each experiment was performed in triplicate.

### 4.7. Halovirus Stability

 After various treatments, samples were removed and diluted in HVD, and virus titres were determined by plaque assay on *Har. hispanica*. Each experiment was performed in triplicate, and representative data are shown. Chloroform sensitivity was examined by the exposure of PH1 lysates in 18% (w/v) MGM to chloroform in a volume ratio of 1 : 4 (chloroform to lysate). Incubation was at room temperature, with constant agitation. At appropriate time points, the mix was allowed to settle, and a sample was removed from the upper layer and diluted in HVD. The effect of a low ionic environment was examined by diluting PH1 lysates (in 18% (w/v) MGM) into double distilled H_2_O in a volume ratio of 1 : 1,000 (lysate to double distilled H_2_O). Incubation was at room temperature, with constant agitation. Samples were removed at various times and diluted in HVD. The pH stability of PH1 was determined by dilution of PH1 lysates in 18% (w/v) MGM in the appropriate pH buffer (HVD buffered with appropriate Tris-HCl) in a volume ratio of 1 : 100 (lysate to buffer). Incubation was at room temperature, with constant agitation. After 30 min, samples were removed and diluted in HVD. Thermal stability of PH1 was examined by a 1 hr incubation of a virus lysate in 18% (w/v) MGM at different temperatures, after which they were brought quickly to room temperature, diluted in HVD, and titrated.

### 4.8. Protein Procedures

 To remove salts, purified virus preparations were mixed with trichloroacetic acid (10% (v/v) final concentration) and incubated on ice for 15 min to allow the proteins to precipitate. After centrifugation (16,000 g, 15 min, room temperature), the precipitate was washed three times in acetone, dried, and resuspended in double distilled H_2_O. Proteins were dissolved in Laemmli sample buffer with 48 mM *β*-mercaptoethanol [50], heated in boiling water for 5 min, then separated on 12% (w/v) NuPAGE Novex Bis-Tris Gels using MES-SDS running buffer, according to the manufacturer's directions (Invitrogen). After electrophoresis, gels were rinsed in double distilled H_2_O and stained with 0.1% (w/v) Brilliant Blue G in 40% (v/v) methanol and 10% (v/v) acetic acid. Gels were destained with several changes of 40% (v/v) methanol and 10% (v/v) acetic acid. Alternatively, gels were stained with GelCode Glycoprotein Staining Kit, Pro-Q Emerald 488 Glycoprotein Gel and Blot Stain Kit, SYPRO Ruby Protein Gel Stain, according to the manufacturer's directions (Invitrogen, Molecular Probes, Pierce Biotechnology). 

 Protein bands were cut from the gels and sent to the Australian Proteome Analysis Facility (Macquarie University) for trypsin digestion and analysis by matrix assisted laser desorption ionisation time of flight mass spectrometry (MALDI-TOF MS) on an Applied Biosystems 4700 Proteomics Analyser (Applied Biosystems).

### 4.9. DNA Procedures

 Proteinase K-treated and nonproteinase K-treated DNA preparations were made from purified PH1 using the methods described previously for SH1 [8]. DNA was separated on 1% (w/v) agarose gels in Tris-acetate-EDTA electrophoresis buffer and was stained with ethidium bromide (Sigma-Aldrich).

*λ* DNA, DNase I (RNase-free), exonuclease III, mung bean nuclease, nuclease BAL-31, T4 DNA polymerase, T4 DNA ligase, T7 exonuclease, and type II restriction endonucleases were purchased from New England Biolabs. RNase A and yeast transfer RNA were purchased from Sigma-Aldrich. Proteinase K was purchased from Promega. Oligonucleotide primers were purchased from Geneworks, Australia. 

To clone fragments of the PH1 genome, purified virus DNA was digested either with *Acc*I, *Eco0109*I, and *Mse*I or *Sma*I restriction endonucleases, blunted with DNA Polymerase I, Large (Klenow) Fragment where appropriate, and ligated into *Acc*I-, *Eco0109*I-, or *Sma*I-digested pBluescript II KS+ using T4 DNA ligase, according to the manufacturer's instructions (New England Biolabs). The DNA was introduced into *E. coli *XL1-Blue and transformants grown on Luria agar containing 15 *μ*g/mL tetracycline and 100 *μ*g/mL ampicillin. The resulting clones were sequenced, and the sequences were used to design specific oligonucleotide primers for PCR amplification and/or primer walking using the virus genome (or specific restriction fragments) as templates.

 To amplify PH1 nucleic acid, approximately 10 ng virus DNA was combined with 500 nM primers, 100 *μ*M of each dNTP, 1 U Deep Vent DNA Polymerase (New England Biolabs), and 1 × ThermoPol buffer (New England Biolabs), in a total reaction volume of 50 *μ*L. Template was denatured at 95°C for 10 min, followed by 30 cycles of denaturation at 95°C for 30 sec, annealing at 56°C for 30 sec, extension at 75°C for 2 min, and a final extension at 75°C for 10 min. PCR reactions were performed on a PxE0.2 Thermo Cycler (Thermo Electro Corporation). Sequencing reactions using 3.2 pmol primer were performed by the dideoxy chain termination method using ABI PRISM Big Dye Terminator Mix version 3.1 on an ABI 3100 capillary sequencer (Applied Biosystems) by the Applied Genetic Diagnostics Sequencing Service at the Department of Pathology (The University of Melbourne).

To obtain terminal genomic fragments, PH1 DNA was digested by the enzyme Pas1, and the head (~700 bp) and the tail (~1300 bp) fragments were purified by the QIAEX II gel extraction kits and treated with 0.5 M piperidine for 2 hr at 37°C to remove protein residues. The piperidine-treated fragments were sequenced by the Genomics Biotech Company (Taipei, Taiwan), using two primers: TGACCAATTAATTAGGCCGGTTCGCC (PH1 head-R) and GTGCCATACTGCTACAATTCT (PH1 tail-F).

Four hundred fifty-four whole genome sequencing: PH1 DNA samples (~5 *μ*g) were sequenced using parallel pyrosequencing on a Roche 454 Genome Sequencer System at Mission Biotech (Taipei, Taiwan). The largest contig was 27399 with 8803 reads (Newbler version 2.7), with 27387 positions being of Q40 quality. The average depth of coverage for each base was 82.9. The GenBank accession for the entire PH1 sequence is KC252997.

## Supplementary Material

This contains a summary table of virus yields throughout a typical purification of halovirus PH1 (supp. Table 1); a gene diagram of a ViPRE (virus and plasmid-related elements) locus found in Hmc. mukohataei (supp. Figure 1), and a nucleotide alignment of the ITRs (inverted terminal repeats) in the genomes of haloviruses PH1, SH1 and HHIV-2 (supp. Figure 2).

## Figures and Tables

**Table 1:** Strains used in this study.

Species/isolate	Strain	Reference
CSW 2.09.4	Original isolate	[51]
*Haloarcula hispanica *	ATCC 33960	[52]
*Haloarcula marismortui *	ATCC 43044	[53]
*“Haloarcula sinaiiensis” *	ATCC 33800	[54]
*Halobacterium salinarum *	NCIMB 763	[55]
*Haloferax gibbonsii *	ATCC 33959	[52]
*Haloferax lucentense *	NCIMB 13854	[56]
*Haloferax mediterranei *	ATCC 33500	[54]
*Haloferax volcanii *	ATCC 29605	[57]
*Halorubrum coriense *	ACAM 3911	[58]
*Halorubrum lacusprofundi *	ACAM 34	[59]
*Halorubrum saccharovorum *	NCIMB 2081	[60]
*Haloterrigena turkmenica *	NCIMB 784	[61]
*Natrialba asiatica *	JCM 9576	[62]

**Table 2:** PH1 virus proteins identified by mass spectroscopy of tryptic peptides.

Protein^a^	Locus tag	ORF	MW (kDa)	Matching peptide masses^b^
			Observed (calculated)	
VP1	HhPH1_gp12	12	185 (158)	16
VP2	HhPH1_gp24	24	100 (78)	15
VP3	HhPH1_gp28	28	40 (38)	5
VP4	HhPH1_gp21	21	35 (26)	5
VP7	HhPH1_gp20	20	24 (20)	5
VP9	HhPH1_gp27	27	16 (17)	3
VP10	HhPH1_gp26	26	15 (17)	4
VP12	HhPH1_gp19	19	7 (9.9)	4

^a^Virus capsid proteins are numbered according to their similarity with SH1 capsid proteins.

^b^The number of peptide masses between 750.0 and 3,513.0 *m/z* identified by MALDI-TOF MS that correspond to the theoretical tryptic peptides of the predicted virus protein.

**Table 3:** Digestion of the PH1 genome by nucleases^a^.

Nuclease (amount)	Proteinase K-treated		Untreated	
	Control	PH1 DNA	Control	PH1 DNA
DNase I (RNase-free) (100 U)	+^b^	+	+^b^	+
Exonuclease III (100 U)	+^b^	+	+^b^	+
Mung bean nuclease (10 U)	+^c^	−	+^c^	−
Nuclease BAL-31 (1 U)	+^b^	+	+^b^	−
RNase A (5 *μ*g/mL)	+^d^	−	+^d^	−
T7 exonuclease (10 U)	+^b^	+	+^b^	−

^a^Extracted nucleic acid from purified PH1 virus was treated with various nucleases and then analysed by gel electrophoresis to detect whether the nuclease digested (+) or did not digest (−) the genome. Control nucleic acids used to confirm activity of the nucleases were  ^b^ *λ* DNA,  ^c^a DNA oligonucleotide, and  ^d^yeast transfer RNA.

**Table 4:** ORF annotations of the halovirus PH1 genome.

ORF^a^	Position^b^	Locus tag	Length^c^ (aa)	pKi	Similarity/characteristics
1	441–605	HhPH1_gp01	50	4.3	76% aa similarity to SH1 ORF2 (YP_271859)
2	602–802	HhPH1_gp02	66	10.9	80% aa similarity to SH1 ORF3 (YP_271860)
3	802–1059	HhPH1_gp03	85	3.9	85% aa similarity to SH1 ORF4 (YP_271861). 44% similarity to HHIV-2 protein 1
4	1056–1283	HhPH1_gp04	75	3.9	85% aa identity to SH1 ORF5 (YP_271862). Predicted coiled-coil region.
5	1276–1686	HhPH1_gp05	136	4.9	80% aa similarity to SH1 ORF6 (YP_271863). Two transmembrane domains. Also related to HHIV-2 protein 2 (38%, AFD02283) and to *Halobiforma lacisalsi* ORF ZP_09950018 (35%)
6	1683–1829	HhPH1_gp06	48	6.1	81% aa similarity to SH1 ORF7 (YP_271864). Predicted signal sequence (SignalP).
7	1822–2130	HhPH1_gp07	102	5.8	91% aa similarity to SH1 ORF8 (YP_271865) and 86% to HHIV-2 putative protein 3 (AFD02284). Other homologs include *Nocardia* protein pnf2110 (YP_122060.1), *Hrr. lacusprofundi* Hlac_0751 (YP_002565421.1), ORFs in actinophage VWB (AAR29707), *Frankia* (EAN11657), and gp40 of *Mycobacterium* phage Dori (AER47690)
8	2123–2383	HhPH1_gp08	86	4.2	90% aa similarity to SH1 ORF9 (YP_271866)
9	2380–2714	HhPH1_gp09	111	5.2	88% aa similarity to SH1 ORF11 (YP_271868) and also similarity to putative protein 4 of HHIV-2 (AFD02285) HlacAJ_19877 of *Hbf. lacisalsi* AJ5 (ZP_09950016)
10	2861–3004	HhPH1_gp10	47	10.4	Weak similarity to SH1 ORF12 (YP_271869) only over the N-terminal 18 residues
11	3029–3100	HhPH1_gp11	23	12.3	Not present in SH1 or HHIV-2
12	3134–7453	HhPH1_gp12	1439	4.2	*Capsid protein VP1.* 72% similarity to SH1 VP1 (ORF 13, YP_271870); HHIV-2 VP1 (AFD02286); HlacAJ_19872 *Hbf. lacisalsi* AJ5. Predicted helix-turn-helix and RuvA-like domain (InterproScan)
13	7505–8227	HhPH1_gp13	240	5.4	*Predicted P-loop ATPase* domain (COG0433). 92% aa similarity to SH1 ORF17 (YP_271874) and also similarity to putative ATPase of HHIV-2 (AFD02288); ATPase of *Haladapatus paucihalophilus* DX253 (ZP_08046180); HlacAJ_19867 of *Hbf. lacisalsi* (ZP_09950014)
14	8419–8228c	HhPH1_gp14	63	5.1	43% aa similarity to SH1 ORF18 (YP_271875). Alanine-rich. Central transmembrane domain (Phobius)
15	8856–8416c	HhPH1_gp15	146	4.1	92% aa similarity to SH1 ORF19 (YP_271876) and also to HHIV-2 putative protein 9 (AFD02290); HlacAJ_19857 of *Hbf. lacisalsi* AJ5; ZOD2009_19093 of *Hap. paucihalophilus *
16	8853–9500c	HhPH1_gp16	215	3.9	65% aa similarity to SH1 ORF20 (YP_271877) and also to putative protein 11 of HHIV-2 (AFD02292); ZOD2009_19098 of *Hap. paucihalophilus; *HlacAJ_19852* Hbf. lacisalsi *AJ5. C-terminal transmembrane domain (Phobius)
17	9500–9919c	HhPH1_gp17	139	4.1	79% aa similarity to SH1 ORF21 (YP_271878) and also to putative protein 12 of HHIV-2 (AFD02293); hypothetical protein HlacAJ_19847 of *Hbf. lacisalsi* (ZP_09950010). Transmembrane domain (Phobius)
18	9923–10594c	HhPH1_gp18	223	4.3	83% aa similarity to SH1 ORF22 (YP_271879) and also to putative protein 13 of HHIV-2 (AFD02294), ZOD2009_19108 of *Hap. paucihalophilus*; HlacAJ_19842 of *Hbf. lacisalsi*. Predicted signal sequence (signalP). Contains 4 CxxC motifs.
19	10659–10943	HhPH1_gp19	94	10.4	*Capsid protein VP12* (ORF19). 91% aa similarity to SH1 VP12 (ORF23, YP_271880) and also to VP12 of HHIV-2 (AFD02295) and HlacAJ_19837 of *Hbf. lacisalsi*; ZOD2009_19113 of *Hap. paucihalophilus*. Two transmembrane domains (Phobius)
20	10960–11517	HhPH1_gp20	185	4.4	*Capsid protein VP7* (ORF20). 98% aa similarity to SH1 VP7 (ORF24, YP_271881) and also to VP7 of HHIV-2 (AFD02296); ZOD2009_19118 of *Hap. paucihalophilus*; HlacAJ_19832 of *Hbf. lacisalsi* AJ5
21	11519–12217	HhPH1_gp21	232	4.1	*Capsid protein VP4* (ORF21). 94% aa similarity to SH1 ORF25 (YP_271882) and also to VP4 of HHIV-2 (AFD02297); ZOD2009_19123 of *Hap. paucihalophilus*; HlacAJ_19827 of *Hbf. lacisalsi* AJ5
22	12233–12454	HhPH1_gp22	73	3.9	81% aa similarity to SH1 ORF26 (YP_271883) and also to putative protein 17 of HHIV-2 (AFD02298);
23	12458–12697	HhPH1_gp23	79	4.8	78% aa similarity to SH1 *capsid protein VP13* (ORF27, YP_271884) and also to VP13 of HHIV-2 (AFD02299). Predicted coil-coil domain. Predicted C-terminal transmembrane domain (Phobius).
24	12701–15064	HhPH1_gp24	787	3.9	*Capsid protein VP2 (ORF24).* 75% aa similarity to SH1 VP2 (ORF28, YP_271885) and to VP2 of HHIV-2 (AFD02300); ZOD2009_19133 of *Hap. paucihalophilus* (ZP_08046189).
25	15065–15961	HhPH1_gp25	298	4.5	*Putative capsid protein VP5* (ORF25). 81% aa similarity to SH1 VP5 (ORF29, YP_271886) and to HHIV-2 VP5 (AFD02301); HlacAJ_19807 of *Hbf lacisalsi* AJ5; ZOD2009_19133 of *Hap. paucihalophilus*.
26	15964–16446	HhPH1_gp26	160	4.6	73% aa similarity to SH1 *capsid protein VP10* (ORF30, YP_271887) and to VP10 of HHIV-2 (AFD02302); ZOD2009_19138 of *Hap. paucihalophilus*; HlacAJ_19802 of *Hbf. lacisalsi*. Predicted C-terminal transmembrane domain (TMHMM).
27	16446–16895	HhPH1_gp27	149	4.2	*Capsid protein VP9* (ORF27). 94% aa similarity to SH1 VP9 (ORF31, YP_271888) and to ZOD2009_19143 of *Hap. paucihalophilus* (ZP_08046191)
28	16908–17921	HhPH1_gp28	337	4.3	*Capsid protein VP3* (ORF28). 91% aa similarity to SH1 VP3 (ORF32, YP_271889) and to NJ7G_2365 of *Natrinema* sp. J7-2
29	17928–18617	HhPH1_gp29	229	4.2	*Capsid protein VP6* (ORF29). 83% aa similarity to SH1 VP6 (ORF33, YP_271890). Also to NJ7G_3194 of *Natrinema* sp. J7-2 and Hmuk_0476 of *Halomicrobium mukohataei*. Predicted carboxypeptidase regulatory-like domain (CarboxypepD_reg, pfam13620)
30	18614–18943	HhPH1_gp30	109	4.9	71% aa similarity to SH1 ORF34 (YP_271891) and to putative protein 27 of HHIV-2 (AFD02308); Predicted signal sequence (signalP)
31	19054–19176c	HhPH1_gp31	40	5.0	Two CxxC motifs. No homolog in SH1 or HHIV-2
32	19173–19289c	HhPH1_gp32	38	4.1	68% aa similarity to SH1 ORF37 (YP_271894)
33	19286–19552c	HhPH1_gp33	88	5.3	58% aa similarity to SH1 ORF39 (YP_271896) and to HHIV-2 putative protein 29 (AFD02310). Four CxxC motifs
34	19549–19731c	HhPH1_gp34	60	7.0	Contains CxxC motif. No homolog in SH1 or HHIV-2
35	19728–20219c	HhPH1_gp35	163	5.6	93% aa similarity to SH1 ORF 41 (YP_271898) and to putative protein 30 of HHIV-2 (AFD02311)
36	20216–21415c	HhPH1_gp36	399	5.0	95% aa similarity to SH1 ORF 42 (YP_271899) and to putative protein 31 of HHIV-2 (AFD02312)
37	21419–21778c	HhPH1_gp37	119	4.1	91% aa similarity to SH1 ORF43 (YP_271900) and to putative protein 32 of HHIV-2 (AFD02313)
38	21762–23006c	HhPH1_gp38	414	4.2	83% aa similarity to SH1 ORF44 (YP_271901) and to putative protein 33 of HHIV-2 (AFD02314). Predicted coil-coil and helix-turn-helix domains
39	23003–23158c	HhPH1_gp39	51	6.5	69% aa similarity to SH1 ORF45 (YP_271902)
40	23161–23370c	HhPH1_gp40	69	3.5	74% aa similarity to SH1 ORF46 (YP_271903), and 55% similarity to putative protein 35 of HHIV-2 (AFD02316)
41	23422–23586c	HhPH1_gp41	54	7.6	86% aa similarity to SH1 ORF47 (YP_271904); Contains 2 CxxC motifs, and shows similarity to protein domain family PF14206. Arginine-rich
42	23583–24023c	HhPH1_gp42		4.8	77% aa similarity to SH1 ORF48 (YP_271905) and to putative protein 36 of HHIV-2 (AFD02317); Hham1_14540 of *Hcc. hamelinensis* (ZP_11271999); PhiCh1p72 of *Natrialba* phage PhiCh1 (NP_665989). DUF4326 (pfam14216) family domain
43	24020–24538c	HhPH1_gp43	172	4.7	79% aa similarity to SH1 ORF49 (YP_271906), and 56% similarity to putative protein 37 of HHIV-2 (AFD02318)
44	24535–25053c	HhPH1_gp44	172	4.3	92% aa similarity to SH1 ORF50 (YP_271907), and 72% similarity to putative protein 38 of HHIV-2 (AFD02319)
45	25050–25277c	HhPH1_gp45	75	4.3	No homolog in SH1 or HHIV-2.
46	25274–25387c	HhPH1_gp46	37	4.8	No homolog in SH1 or HHIV-2
47	25533–25904	HhPH1_gp47	123	4.3	61% aa similarity to SH1 ORF51 (YP_271908); 66% similarity to putative protein 39 of HHIV-2 (AFD02320). Predicted COG1342 domain (DNA binding/helix-turn-helix)
48	25901–26092c	HhPH1_gp48	63	4.9	Contains CxxC motif. No homolog in SH1 or HHIV-2
49	26089–27648c	HhPH1_gp49	506	4.0	81% aa similarity to SH1 ORF55 (YP_271912) (but only in the N-terminal half). Homolog of *virus structural protein VP18* of HHIV-2 (AFD02323)

^a^ORFs were predicted either by GLIMMER or by manual searching for homologs in the GenBank database.

^b^Start and end positions of ORFs are give in bp number according to the PH1 sequence deposited at GenBank (KC252997). ORFs on the complementary strand are denoted by the suffix c.

^c^Length of the predicted ORF, in number of amino acids.

**Table 5:** Main differences (divergent regions) between the SH1 and PH1 genomes.

Region^a^	SH1 start^b^	SH1 stop	Length (bp)	PH1 start	PH1 stop	Length (bp)	Comment
DV1	527	650	123	538	604	66	Replacement. Both regions have direct repeats at their borders; PH1 has two sets GACCCGGC and CGCTGC, while SH1 has CCCGAC. In SH1, this replacement region includes the C-terminal region of ORF2 and the N-terminal region of ORF3. In PH1, it covers only the C-terminal region of ORF1

DV2	2433	2588	155	2386	2387	—	RMD^c^ from PH1. SH1 repeat at border is TGACCG. This removes the homolog of SH1 ORF10 from PH1

DV3	3324	3598	274	3137	3416	279	Replacement at the beginning of SH1 ORF13/PH1 ORF12 (capsid protein VP1)

DV4	6656	7128	472	6478	7085	607	Replacement near the C-terminus of SH1 ORF13/PH1 ORF12 (capsid protein VP1)

DV5	7491	8341	850	7446	7447	—	Indel that results in ORFs14-16 of SH1 not being present in PH1. SH1 ORF14 has a close homolog in HHIV-2 (ORF6), in a similar position, just after the VP1 homolog. SH1 ORF15 is a conserved protein in haloarchaea (e.g. NP_2552A, and Hmuk_2978) and halovirus His1 (ORF13). Possible inverted repeat (AGCCATG) at border of SH1 region

DV6	13705	13706	—	12818	12851	33	RMD from SH1. PH1 repeat at border is CAGCGG(g/t)G. This removes a part of capsid protein VP2 sequence from the SH1 protein

DV7	13789	13863	74	12934	12941	7	Replacement. This occurs within VP2 gene of both viruses

DV8	15142	15186	44	14220	14221	—	RMD from PH1. SH1 repeat at border is TG(t/c)CCGACGA and occurs within capsid protein VP2 gene of both viruses

DV9	15303	15317	14	14337	14338	—	RMD from PH1. SH1 repeat at border is GCCGACGA and occurs within capsid protein VP2 gene of both viruses

DV10	15504	15527	23	14529	14530	—	RMD from PH1. SH1 repeat at border is GACGA and occurs within capsid protein VP2 of both viruses

DV11	20026	20405	379	19019	19173	154	Replacement beginning at the start of SH1 ORF35/PH1 ORF31. This region is longer in SH1, where it includes ORF36, an ORF that has no homolog present in PH1

DV12	20440	20661	221	19208	19286	78	Replacement. This is unequal and includes an ORF in SH1 (ORF38) that is not present in PH1

DV13	20943	21085	142	19562	19728	166	Replacement. This covers SH1 ORF40/PH1 ORF34. The predicted proteins are not homologous

DV14	23541	23542	—	22171	22197	27	Probable RMD in SH1. PH1 repeat at border is CGTCTCGG and occurs in SH1 ORF44/PH1 ORF38

DV15	24506	24521	15	23152	23153	—	Probable RMD in PH1. SH1 repeat at border is CTCGGT and occurs near the end of SH1 ORF45/PH1 ORF39

DV16	24951	24964	13	23579	23580	—	Probable RMD in PH1. SH1 repeat at border is CGGTC and occurs in SH1 ORF47/PH1 ORF41

DV17	26449	26450	—	25050	25274	224	Probable RMD in SH1. PH1 repeat at border is TCATGCG and occurs near the start of SH1 ORF50/PH1 ORF44 and provides an extra ORF for PH1 (ORF45)

DV18	27074	29764	690	25895	26933	1038	Replacement. Left border at start of SH1 ORF51/PH1 ORF47 and extends rightwards into SH1 ORF55/PH1 ORF49 (VP18 gene). It is an unequal replacement and SH1 has two more ORFs in this region than PH1

^a^DV: Divergent regions between the genomes of SH1 and PH1.

^b^Start and stop positions refer to the GenBank sequences of the two viruses: SH1, NC_007217.1; PH1, KC252997.

^c^RMD: repeat-mediated deletion event, as described in [20].

**Table 6:** Transfection of haloarchaea by PH1 DNA.

Species^a^	Efficiency of transfection/transformation with	
	PH1 DNA^b^ (PFU/*μ*g of DNA)	pUBP2 DNA^b,c^ (CFU/*μ*g of DNA)
*Har. hispanica *	5.3 ± 0.5 × 10^3^	1.4 ± 0.8 × 10^4^
*Har. marismortui *	4.5 ± 0.7 × 10^3^	1.8 ± 0.1 × 10^4^
*“Har. sinaiiensis” *	3.2 ± 0.6 × 10^2^	—
*Halobacterium salinarum *	—	5.4 ± 4.2 × 10^3^
*Haloferax gibbonsii *	2.8 ± 0.8 × 10^3^	4.0 ± 2.6 × 10^3^
*Haloferax lucentense *	—	5.7 ± 1.1 × 10^3^
*Hfx. volcanii *	—	2.1 ± 0.7 × 10^5^
*Hrr. lacusprofundi *	7.0 ± 2.2 × 10^2^	9.5 ± 1.5 × 10^3^
*Haloterrigena turkmenica *	1.6 ± 1.7 × 10^2^	2.4 ± 0.6 × 10^3^
*Natrialba asiatica *	3.5 ± 3.9 × 10^2^	3.1 ± 1.0 × 10^4^

^a^Only those species positive for transfection and/or plasmid transformation are shown.

^b^Rates of transfection by (nonprotease treated) PH1 DNA or transformation by plasmid pUB2 are averages of three independent experiments, each performed in duplicate (± standard deviation).

^c^Transformants were selected on plates with 2, 4, or 6 *μ*g/mL simvastatin, depending on the strain.

— no plaques or colonies observed.
