# Supplementary material for: PH1: An Archaeovirus of Haloarcula hispanica Related to SH1 and HHIV-2
Source: Archaea. 2013 Mar 21;2013:456318. doi: 10.1155/2013/456318 (PMC3622292; doi:10.1155/2013/456318)

##### Supplementary Table 1. Typical yields of PH1 virus during purification.

| **Purification Step**^a^ | **Infectivity**  **(Total PFU)** | **% Recovery of infectivity** | **Specific infectivity**  **(PFU/*A*_260_)** |
| --- | --- | --- | --- |
| 1. Infected cell supernatant | 1.9 × 10^13^ | 100 | NA |
| 2. Pelleted virus | 1.9 × 10^13^ | 100 | NA |
| 3. Sucrose gradient | 4.4 × 10^12^ | 23 | 5.8 × 10^10^ |
| 4. CsCl gradient | 1.5 × 10^12^ | 8 | 4.5 × 10^11^ |

^a^ See text for details of each step

Supplementary **Figure 1.** ViPRE _Hmuk1_ of *Hmc. mukohataei* (nt 424105 – 463483 of NC_013202) showing the relationships of these genomic ORFs to the ORFs of haloviruses. For orientation, the locus tags of several *Hmc. mukohataei* ORFs are given below them, and a scale in kb is shown at the bottom level. ORFs coloured black are integrase or ORC1 homologs; red ORFs represent His2/pleolipovirus capsid protein homologs; light blue ORFs are similar to those of halovirus SNJ1; green ORFs are related to ViPRE _Hmar1_ of *Haloarcula marismortui* (see text). Other labels are: *int*, integrase; PL6, a homolog of the *Hqr. walsbyi* plasmid PL6A gene Hqrw_6002; ΦH-r, phiH-like repressor; glycos, probable DNA glycosylase; MTase, putative DNA methyltransferase; halo-Φ, halovirus related gene; tRNA-ala, transfer RNA (alanine); *att*, probable integration site, and a direct repeat of part of the tRNA-ala gene seen at the right end.


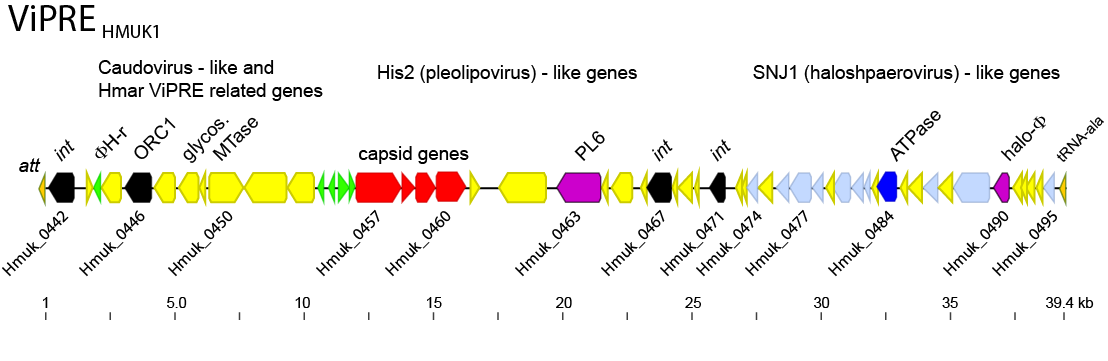


Supplementary **Figure 2**. CLUSTAL alignment of the ITRs of PH1, SH1 and HHIV-2. The ITRs were aligned using CLUSTALW 2.1. The viruses are indicated by labels at the left of each sequence. Asterisks indicate bases that are conserved in all three viruses. Numbered regions in brackets highlight conserved regions between the three sequences.


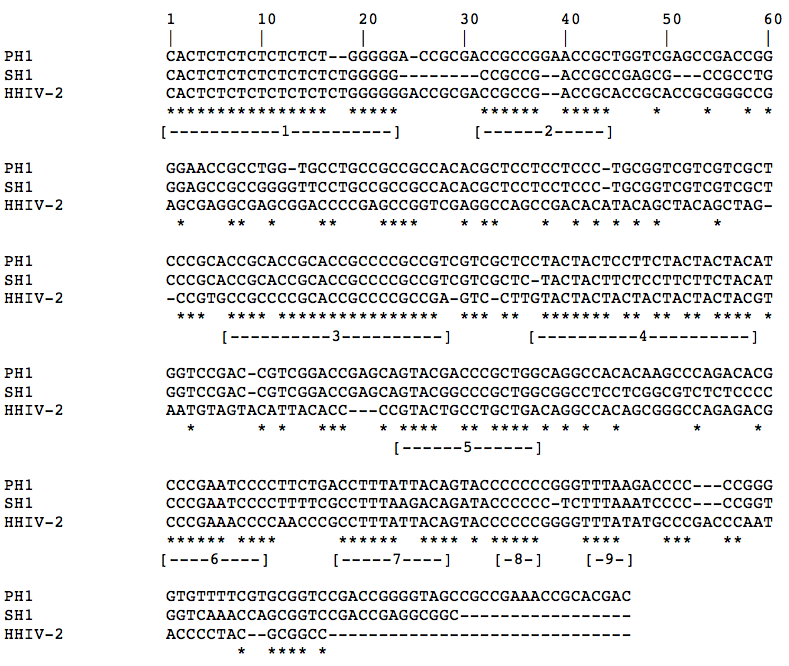

Supplement: Supplementary file 1 — This contains a summary table of virus yields throughout a typical purification of halovirus PH1 (supp. Table 1); a gene diagram of a ViPRE (virus and plasmid-related elements) locus found in Hmc. mukohataei (supp. Figure 1), and a nucleotide alignment of the ITRs (inverted terminal repeats) in the genomes of haloviruses PH1, SH1 and HHIV-2 (supp. Figure 2). [file 456318.f1.doc]
